# Effect of routine extradural optic canal decompression performed by skull base trained surgeons on visual outcomes in patients with anterior skull base meningiomas

**DOI:** 10.1007/s00701-025-06584-7

**Published:** 2025-06-16

**Authors:** Yasmin Sadigh, Lailla Talbi, Juliette Monchen, Ayca Cozar, Kelsey Gori, Eelke M. Bos, Ruben Dammers, Victor Volovici

**Affiliations:** 1 Department of Neurosurgery, Erasmus MC Stroke Center, Erasmus University Medical Centre, Dr Molewaterplein 40, Rotterdam, 3015 GD The Netherlands; 2 Centre for Complex Microvascular Surgery, Erasmus University Medical Centre, Rotterdam, The Netherlands; 3 Brain Tumor Center, Erasmus MC Cancer Institute, Erasmus University Medical Centre, Rotterdam, The Netherlands

**Keywords:** Extradural anterior clinoidectomy, Optic canal decompression, Visual acuity, Skull base surgery

## Abstract

**Purpose:**

Optic canal decompression is a surgical option in anterior skull base tumors with optic nerve involvement. Meningiomas may grow into the optic canal even without evidence of involvement on MRI studies. We aim to investigate the effect of routine optic canal unroofing performed by skull base trained surgeons versus general neurosurgeons on the postoperative visual outcomes in anterior skull base meningiomas.

**Methods:**

Between January 2013 and October 2023, consecutive patients in our institution who underwent craniotomies due to visual impairment were retrospectively reviewed. Patient records were screened for data on optic nerve compression, patient characteristics, lesion characteristics, intraoperative factors, the exact preoperative and postoperative visual acuity, as well as the postoperative clinical course. The primary outcome was the change in visual acuity postoperatively compared to the preoperative visual acuity. Multivariable linear regression analysis was performed with best postoperative visual acuity as a dependent adjusting for prognostic factors.

**Results:**

Out of 709 patients who underwent craniotomies for anterior skull base meningiomas, 94 patients showed optic nerve involvement on MRI. In total, 59 cases were treated by skull base trained surgeons and 35 by general neurosurgeons. Optic canal decompression was performed in 65% of the patients. There was no significant difference between patients treated by skull base surgeons and general neurosurgeons in terms of postoperative permanent complications. In patients with tuberculum sellae or anterior clinoid process meningiomas, postoperative secondary deterioration of visual acuity occurred in 40% (*n* = 10) of the cases treated by general neurosurgeons versus 11% (*n* = 4) in the group treated by skull base trained surgeons. In cases with a preoperative visual acuity of 0.2 or lower (35%, *n* = 33), 42% (*n* = 14) reached a best postoperative visual acuity of 0.5 or higher. Nineteen (20%) cases presented with functional blindness preoperatively. Of these, nine (47%) cases showed significant vision improvement postoperatively. Multivariable linear regression analysis revealed that patients with higher preoperative visual acuity reached a higher best visual acuity postoperatively.

**Conclusion:**

Patients with tuberculum sellae and anterior clinoid process meningiomas benefit from skull base surgeons trained in extradural optic canal decompression, as reflected by lower postoperative secondary visual acuity deterioration in patients treated by skull base trained surgeons. All cases presenting with tumors with optic apparatus involvement should be managed by skull base trained surgeons to maximize postoperative visual acuity preservation.

**Supplementary information:**

The online version contains supplementary material available at 10.1007/s00701-025-06584-7.

## Introduction

Anterior skull base tumors with optic nerve involvement pose a considerable challenge even for experienced skull base surgeons [1]. In general, the optic canal is not opened unless there is a suspicion that the tumor exhibits optic canal invasion. In 1985, Dolenc firstly introduced extradural anterior clinoidectomy (EAC), which was used to gain a better and safer exposure when approaching lesions surrounding the central segment of the carotid artery. [8] Currently, EAC is not only recognized as a neurosurgical technique but also one of the most facilitating methods to optimize surgical exposure in parasellar regions [15].

Previous evidence has shown that EAC and optic canal unroofing is a safe and effective technique for improving or maintaining the visual ability of patients, which could be compromised due to optic nerve compression caused by suprasellar lesions [5, 10, 13, 16]. Harbouring and maintaining surgical expertise in performing EAC is essential due to the critical anatomical surroundings of the anterior clinoid process (ACP).

In the Erasmus MC University Medical Center in Rotterdam, traditionally anterior skull base lesions were not de facto centralised to skull base surgeons alone. In more recent years, owing to a retrospective evaluation and subsequent benchmarked evidence that recurrences were primarily seen arising from the optic canal, a change in clinical practice occurred. Anterior skull base meningiomas with optic nerve involvement were progressively centralised starting in 2013 and after 2017 ACP resection and optic canal unroofing became the standard in all cases. This study aims to investigate whether there is a difference in the postoperative visual outcomes of patients treated by general neurosurgeons without a skull base subspecialty compared to the new paradigm of extradural optic canal decompression by skull base trained surgeons.

## Methods

### Eligibility criteria

All consecutive patients in our institution who underwent craniotomies for anterior skull base lesions due to objective and/or subjective visual impairment between January 2013 and October 2023 were retrospectively reviewed. Patient records were screened for data on optic nerve compression, determined by Magnetic Resonance Imaging (MRI) performed preoperatively and the corresponding radiological report. To be included in this study, data on the exact preoperative and postoperative visual acuity measured by an independent ophthalmologist had to be available. The indication for surgery had to include optic nerve decompression. Patients were excluded if the optic nerve was not compressed (confirmed by MRI and radiological report), if the exact (preoperative and/or postoperative) visual acuity was unavailable, only partial resections without optic nerve decompression were part of the surgical strategy, the surgical report was unavailable, the concerning pathology was of vascular nature, or full orbital exenteration was performed.

General informed consent for the collection of data was granted from every participant upon admittance to the hospital. This study was conducted in accordance with the Declaration of Helsinki. Due to a retrospective study design and as a part of a non-Medical Research Involving Human Subjects Act and no Internal Review Board approval from the Erasmus MC Medical Ethics Review Committee (MERC, METC in Dutch) was needed. Under this agreement, permission was granted to include data from all patients harbouring brain tumors.

### Data collection and outcome definitions

Data were extracted by five independent authors (YS, JM, AC, LT, KG) from the patient records and checked by the first author (YS). Disagreements were resolved through discussion with the senior author (VV).

Baseline characteristics such as gender, age, duration of visual impairment (in months), preoperative visual field status, preoperative visual acuity measured by the department of Ophthalmology, lesion type (tumor, vascular, other), lesion location, lesion size (mm) were extracted.

Surgical data, such as the type of neurosurgeon (skull base trained surgeon, general neurosurgeon) performing the resection, the surgical approach, and whether EAC was performed, were extracted. For meningiomas, Simpson Grade, 2016 WHO Meningioma Tumor Grade, and whether postoperative radiotherapy was performed were extracted. Neurosurgeons were categorized as ‘skull base trained surgeons’ when a skull base fellowship and a high-volume skull base training in the last years of residency was completed, and if they had a minimum of three years of experience as skull base surgeons. Transient postoperative complications were defined as procedure-related transient cranial nerve palsy and/or transient neurological deficits which occurred postoperatively. Permanent postoperative complications were defined as procedure-related permanent cranial nerve palsy and/or permanent neurological deficits which occurred postoperatively. Number of recurrences and time until recurrence (in months) was both extracted. Time until recurrence was defined as the time between the first surgery until first signs of recurrence were observed in months. Period of preoperative blindness was measured in months in patients with a preoperative visual acuity of 0.1 or lower.

Intraoperative factors such as intraoperative blood loss (in mL), duration of surgery (in minutes), vascular damage to the optic nerve, level of involvement of the optic nerve with the tumor, difficulty of tumor resection, and tumor consistency were also extracted.

For postoperative visual outcomes, parameters such as postoperative visual acuity measured by the department of Ophthalmology, change in visual acuity postoperatively, best postoperative visual acuity, and time until best postoperative vision acuity (months) were extracted. Data on secondary postoperative vision deterioration and need of reoperation due to secondary vision impairment were also extracted. Secondary deterioration was defined as vision impairment occurring during the postoperative follow-up. The change in visual field postoperatively compared to the preoperative visual field was stated objectively through the comparison of the preoperative and postoperative Goldmann field exam (performed by independent specialists from the department of Ophthalmology).

The change in visual acuity postoperatively compared to the preoperative visual acuity was stated objectively through the comparison of the preoperative and postoperative ophthalmologic exam. An increase of 0.1 points or more in the postoperative visual acuity was defined as improved vision. A decrease of 0.1 points of more in the postoperative visual acuity was defined as worsened vision. A visual acuity of 1.0 or higher was considered to be intact. Poor postoperative visual outcome was defined as a postoperative visual acuity of 0.1 or lower in patients with a preoperative visual acuity of 0.2 or higher.

### Surgical technique

For every case in which any kind of involvement of the optic nerve is seen on the preoperative MRI, we use a similar approach. The patient is placed supine on the operating table, with the head rotated at 30–45 degrees, depending on the desired angle of approach determined by the senior surgeon. A curvilinear incision starting at the upper edge of the root of zygoma and bending just above the frontozygomatic suture is usually employed. After skin incision and interfascial dissection, the temporalis muscle is freed up from its insertion on the zygoma completely and reflected inferiorly in order to ensure proper superior visualization. After a craniotomy is performed, the greater sphenoid wing is drilled with a 4 mm cutting burr and the periorbit is exposed. The meningo-orbital band is skeletonized free from bone, coagulated and cut. By having the periorbit exposed, one can easily identify the plane between the dura propria and the periosteal dura of the temporal lobe and thus peel the lateral wall of the cavernous sinus until foramen rotundum and V2. In some larger meningiomas, the peeling is extended as far as V3, or even the apex of the petrous bone. This is done by using a Penfield dissector, but also with precise cuts in some areas where the dural layers are more heavily intertwined, such as between V1 and V2. After the dura is peeled posteriorly, the optic canal is identified subfrontally and intraorbitally, and the optic nerve (still protected in its sheet) is exposed. The optic canal roof is drilled using a 2 mm cutting burr and special rongeurs. The final step is to remove the tip of the ACP by first disconnecting it from the optic strut and dissecting the adhesions to the dura and anterior petroclinoid fold.

The dura covering the ACP is fully dissected such that it can be internally decompressed with the burr and special rongeur. The clinoidal part of the carotid artery is dissected after which the tip of the ACP is resected. Preoperatively, 3D-CT reconstruction of this region was performed to investigate the existence of a bony carotid ring, a carotico-clinoid foramen, a middle clinoid process, or pneumatization of the ACP [3, 19, 20].

Once hemostasis is achieved, the optic nerve sheet is incised medially, or far laterally, beginning halfway with an arachnoid knife and fine dissectors. The incision plan depends on the preoperative planning. Care is taken to identify the ophthalmic artery early if the incision is placed laterally. Almost in all cases, tumor is identified inside the optic canal.

### Statistical analysis

Descriptive statistics were used to characterize the baseline characteristics of patients with visual impairment. Shapiro–Wilk test was performed to determine whether the parameters were normally distributed. Normally distributed variables were reported as means and standard deviations (SD). Non-normally distributed variables were reported as medians and interquartile ranges (IQR). Categorical variables were reported as absolute numbers of cases (N) and percentages of the total. Data was stratified for surgeries performed by skull base trained surgeons vs. general neurosurgeons. Groups were compared using the Chi-square test, Fisher’s Exact Test, or Fisher-Freeman-Halton Exact Test for categorial variables and Mann–Whitney U test or One-Way ANOVA for continuous variables. Sensitivity analysis was performed for descriptive statistics and visual outcomes of patients who underwent primary resections.

Univariable linear regression analysis was used to identify candidate variables influencing the postoperative visual outcome for the multivariable linear regression analysis. Variables such as sex, age, duration of preoperative vision impairment, preoperative visual acuity, lesion size, lesion location, type of neurosurgeon, Simpson Grade, optic canal decompression, clinoidectomy, intraoperative blood loss, duration of surgery, vascular damage to the optic nerve, level of involvement of the optic nerve with the tumor, difficulty of tumor resection, and tumor consistency were preselected. Variables with p-values of less than 0.20 derived from the univariable linear regression analysis, were included in the multivariable linear regression analysis. Multivariable linear regression analysis was performed for best postoperative visual acuity as a dependent. Regression Coefficients (β), Standard Errors (SE), and 95% Confidence Interval (CI) were calculated.

Statistical analyses were performed using SPSS software (IBM SPSS Statistics for Windows, Version 29.0.1.0., Armonk, New York). A p-value < 0.05 was considered as statistically significant.

## Results

### Baseline characteristics

**Fig. 1:**
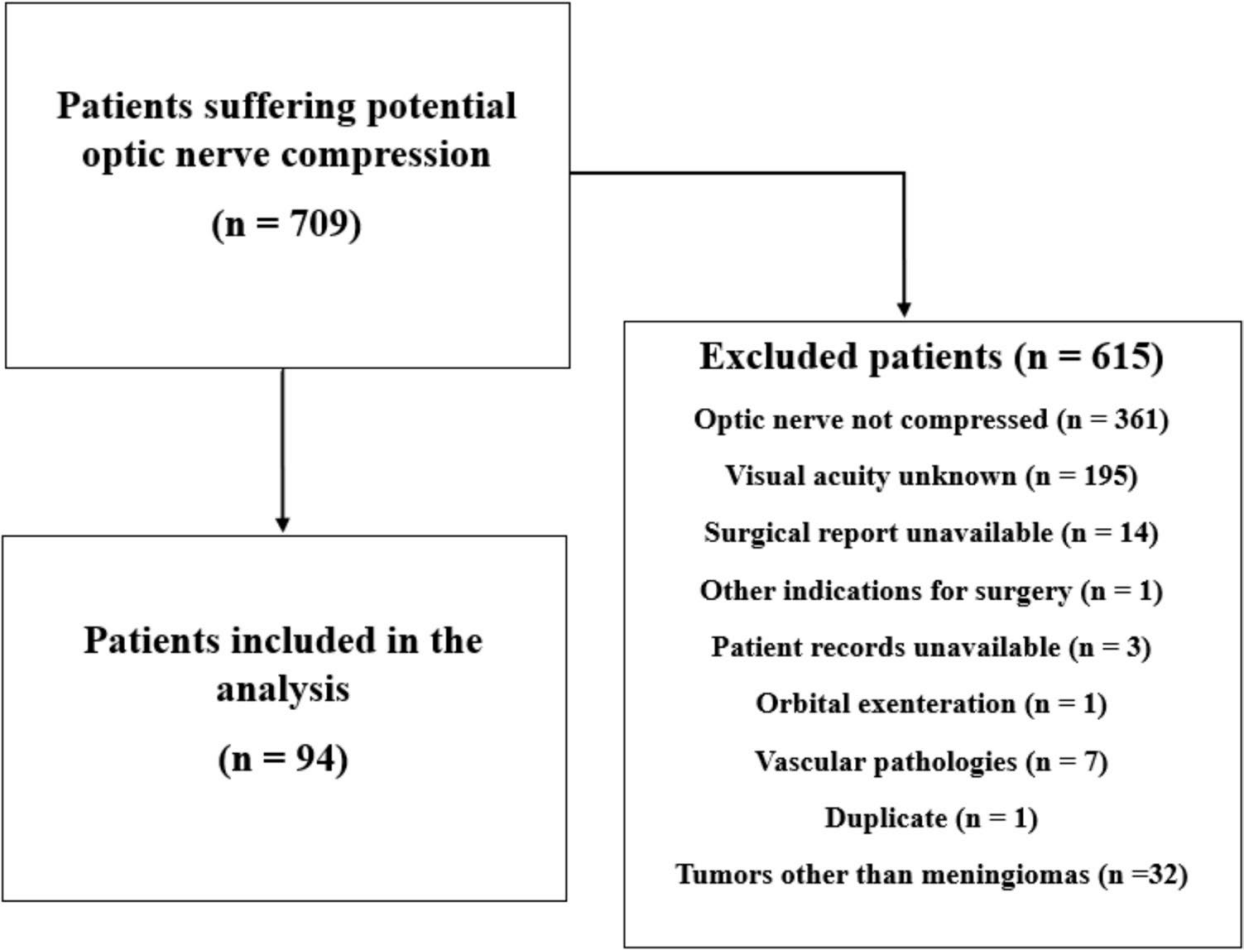
Patient Inclusion Flowchart

Between January 2013 and October 2023, a total of 709 patients underwent craniotomies in our institute for anterior skull base lesions, with or without optic nerve compression (Fig. 1). In 361 patients, the optic nerve was not compressed (confirmed by MRI and radiological report). In 195 patients, preoperative and/or postoperative visual acuity were unavailable. For 14 patients, surgical reports were unavailable. Three patient records were unavailable. One patient was operated due to other surgical indication than optic nerve decompression. In one patient orbital exenteration was performed. Seven patients suffered from intracranial aneurysms with optic nerve compression. Thirty-two patients were excluded from the main analysis based on other pathologies than meningiomas. Ultimately, 94 patients were included in the analysis (Fig. 1, Table 1). All patients underwent primary resections.

**Table 1:** Baseline characteristics of patients treated by skull base trained surgeons vs. general neurosurgeons

	No. of patients (%)			
	All patients	Skull base trained surgeon	General neurosurgeon	*P* value
	(*n* = 94)	(*n* = 59)	(*n* = 35)	
Sex, female	73 (78)	46 (78)	27 (77)	0.93
Age (year), median (IQR)	51 (40.7–62.2)	50 (39–61)	51 (46–66)	0.31
Duration vision impairment (mo), median (IQR)	7 (3–12)	7 (3–12)	6 (3–13.5)	0.92
Preoperative visual field				0.59
Intact	26 (30)	18 (34)	8 (24)	
Homonymous hemianopia	7 (8)	6 (11)	1 (3)	
Bitemporal hemianopia	11 (13)	6 (11)	5 (15)	
Quadrantanopia superior	3 (3)	2 (4)	1 (3)	
Quadrantanopia inferior	7 (8)	3 (6)	4 (12)	
Nasal hemianopsia	4 (5)	3 (6)	1 (3)	
Non-intact (not specified)	28 (33)	15 (28)	13 (39)	
Preoperative visual acuity, median (IQR)	0.4 (0.1–0.8)	0.4 (0.1–0.9)	0.4 (0.2–0.7)	0.26
Lesion location				0.10
Tuberculum sellae	44 (47)	25 (42)	19 (54)	
Anterior clinoid process	23 (24)	17 (29)	6 (17)	
Sphenoid: middle-ridge	9 (10)	7 (12)	2 (6)	
Sphenoid: outer-ridge	3 (3)	2 (3)	1 (3)	
Cavernous sinus	5 (5)	5 (8)	0 (0)	
Fronto-basal convexity	3 (3)	1 (2)	2 (6)	
Other	7 (7)	2 (3)	5 (14)	
Lesion size (mm), median (IQR)	27 (17.2–38)	28 (16–38)	26 (21–40)	0.64

*IQR*; Interquartile Range, *mo*; Months

For the entire cohort, 78% of the patients were women (*n* = 73) (Table 1). The median age was 51 years (IQR 40.7–62.2). The median duration of preoperative visual impairment was seven months (IQR 3–12). Fifty-nine patients were treated by skull base trained surgeons and 35 by general neurosurgeons (Table 1). The median preoperative visual acuity in cases treated by skull base trained surgeons was 0.4 (IQR 0.1–0.8) and in cases treated by general neurosurgeons 0.4 (IQR 0.2–0.7) (p = 0.26). In 47% (*n* = 44) of the patients, the lesion was situated on the tuberculum sellae and in 24% (*n* = 23) of the patients the lesion was situated at the ACP. In cases treated by skull base trained surgeons, the median lesion size was 27 mm (IQR 17.2–38), compared to 26 mm (IQR 21–40) in patients treated by general neurosurgeons (*p* = 0.64).

### Surgical outcomes

Unroofing of the optic canal was performed in 72% of the cases (*n* = 65). EAC was performed in 53% (*n* = 47) of the patients. Postoperative transient complications occurred in 17% of the patients (*n* = 16), with a statistically significant difference between patients treated by skull base trained surgeons versus general neurosurgeons (10%, n = 6 and 29%, *n* = 10; *p* = 0.02, respectively). Postoperative permanent complications occurred in 8% (*n* = 7), with no statistically significant difference between cases treated by skull base surgeons and general neurosurgeons (9%, *n* = 5 and 6%, *n* = 2; *p* = 0.71, respectively). In patients treated by skull base trained surgeons, two (3%) patients suffered from permanent oculomotor nerve palsy, one (2%) patient from trochlear nerve palsy, and three (5%) from facial nerve palsy. In patients treated by general neurosurgeons, one (3%) patient suffered from facial nerve palsy, and one patient (3%) from permanent visual impairment due to optic nerve injury.

Eight (14%) patients treated by skull base trained surgeons showed recurrence compared to five patients treated by general neurosurgeons (14%; *p* = 0.92). Time until recurrence was 31 months (IQR 27.2–54.7) in patients treated by skull base trained surgeons and 36 months (IQR 18.5–70) in patients treated by general neurosurgeons (*p* = 0.94) (Table 2).

**Table 2:** Surgical outcomes of patients treated by skull base trained surgeons vs. general neurosurgeons

	No. of patients (%)			
	All patients	Skull base trained surgeon	General neurosurgeon	*P* value
	(*n* = 94)	(*n* = 59)	(*n* = 35)	
Surgical approach				0.96
Pterional	70 (79)	46 (78)	24 (83)	
Pretemporal	11 (12)	8 (14)	3 (10)	
Fronto-orbital	5 (6)	3 (5)	2 (7)	
Anterior petrosectomy	1 (1)	1 (2)	0 (0)	
Trans-sphenoidal	1 (1)	1 (2)	0 (0)	
Optic canal decompression	65 (72)	42 (72)	23 (72)	0.96
Clinoidectomy	47 (53)	32 (55)	15 (48)	0.54
Simpson Grade Meningioma				0.66
Grade 0	2 (2)	2 (3)	0 (0)	
Grade I	2 (2)	2 (3)	0 (0)	
Grade II	31 (35)	21 (36)	10 (33)	
Grade III	25 (28)	14 (24)	11 (37)	
Grade IV	28 (32)	19 (33)	9 (30)	
Missing Simpson Grade	6	1	5	
Postoperative transient complications	16 (17)	6 (10)	10 (29)	0.02
Postoperative permanent complications	7 (8)	5 (9)	2 (6)	0.71
Meningioma WHO Tumor Grade				0.49
Grade I	84 (89)	54 (91)	30 (86)	
Grade II	10 (11)	5 (9)	5 (14)	
Postoperative radiotherapy	6 (6)	5 (8)	1 (3)	0.41
No. of recurrences	13 (14)	8 (14)	5 (14)	0.92
Time until recurrence (mo), median (IQR)	34 (25–58)	31 (27.2–54.7)	36 (18.5–70)	0.94

*IQR*; Interquartile Range, *mo*; Months, *WHO*; World Health Organization

**Fig. 2:**
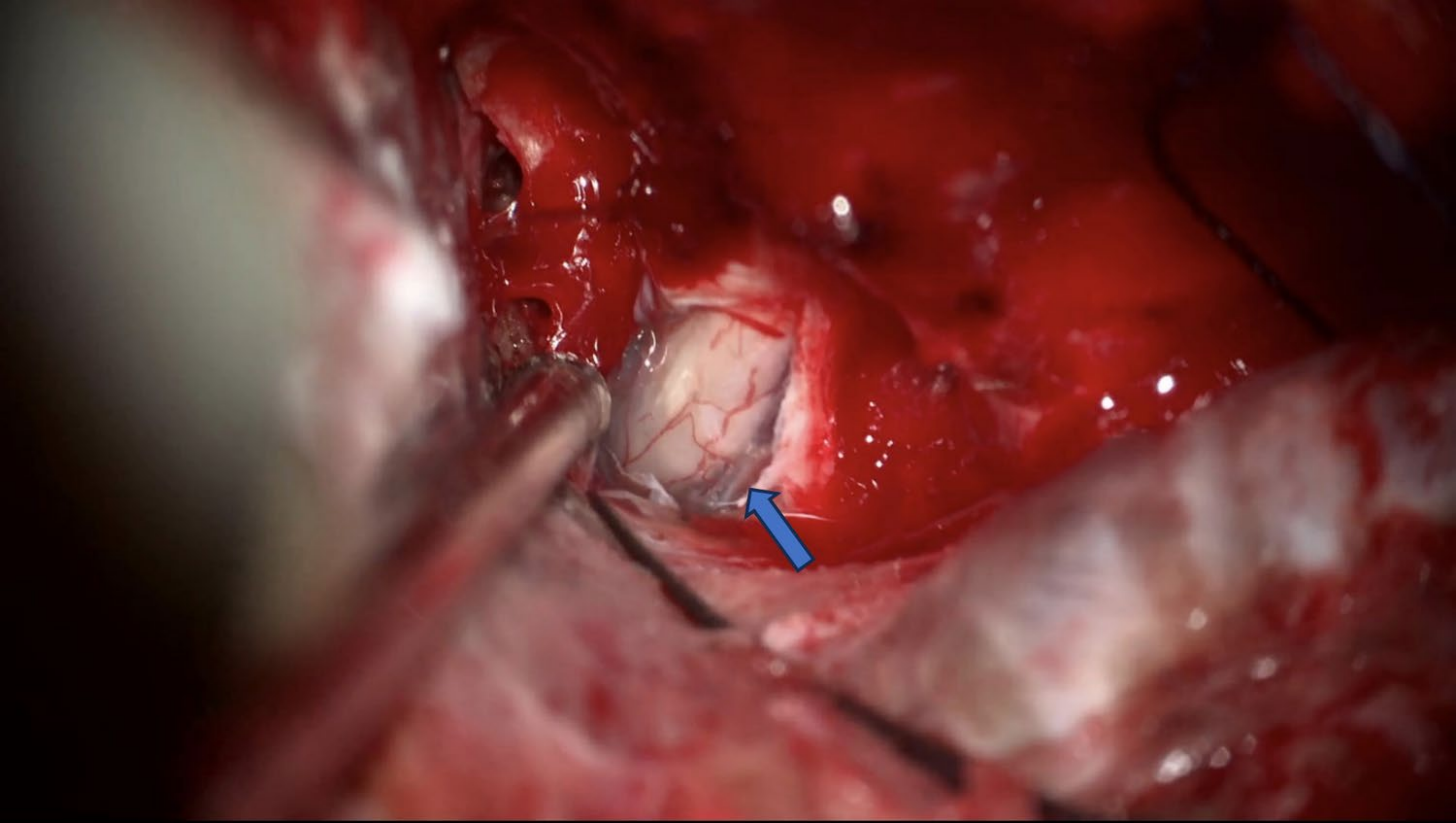
**Perioperative image of optic nerve involvement**. Right-sided optic nerve, note the open canal, opened canal dura overlying the opticus and the tumor latero-inferior (blue arrow) to the nerve, despite no evidence of canal involvement on MRI

**Fig. 3:**
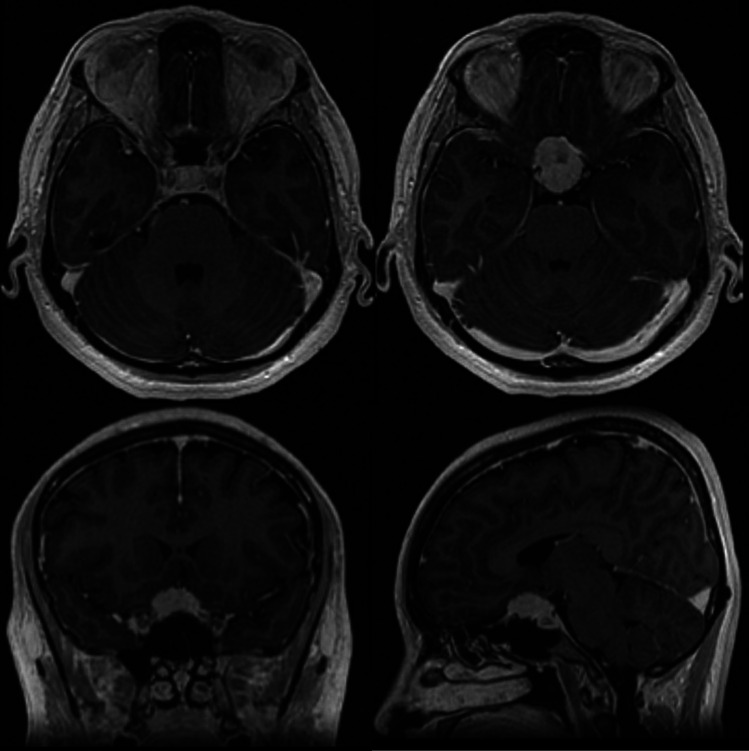
**Preoperative MRI of a case with a tuberculum sellae meningioma**. No tumor infiltration of the optic canal is visible. However, intraoperatively, tumor invasion inside the optic canal was found

**Fig. 4:**
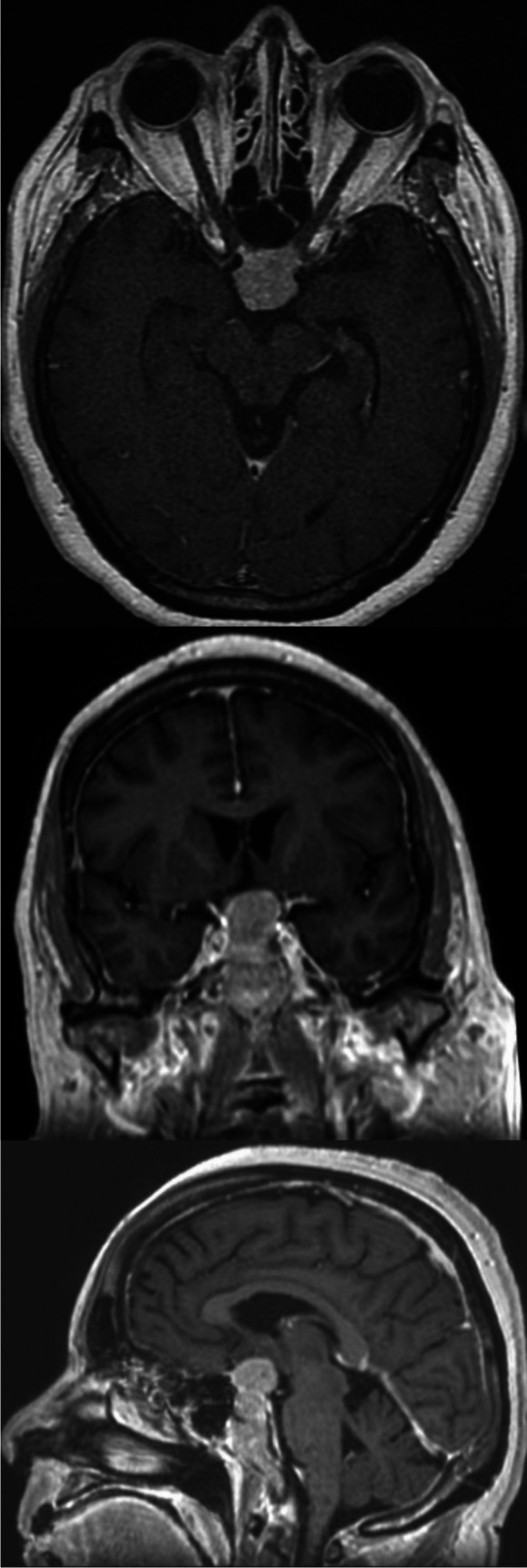
**Optic apparatus has been affected by the tumor**: No tumor infiltration of the optic canal is visible. However, intraoperatively, tumor invasion inside the optic canal was found

In all meningioma cases where the optic canal was unroofed, tumor was found encroached around the optic nerve, even in cases where MRI did not suggest growth in the optic canal (Figs. 2, 3, and 4).

### Visual outcomes

In patients treated by skull base trained surgeons, 50 patients experienced preoperative impaired vision and in nine patients the preoperative vision was intact (Table 3). General neurosurgeons treated only cases that experienced preoperative visual impairment (*n* = 35). In patients treated by skull base trained surgeons, 35 patients experienced preoperative impaired visual field and in eighteen cases preoperative visual field was intact (Table 3). General neurosurgeons treated 25 patients with preoperative visual field impairment and eight cases with preoperative intact visual field.

**Table 3:** Postoperative visual outcomes of patients treated by skull base trained surgeons vs. general neurosurgeons

	No. of patients (%)				
	Skull base trained surgeon		General neurosurgeon		*P* value
	(*n* = 59)		(*n* = 35)		
	Preoperative impaired vision	Preoperative intact vision	Preoperative impaired vision		
	(*n* = 50)	(*n* = 9)	(*n* = 35)		
Postoperative visual acuity, median (IQR)	0.5 (0.09–1.0)	1.0 (1.0–1.0)	0.5 (0.01–0.9)		0.37
Postoperative visual acuity					0.60
Same as preoperative	12 (24)	-	13 (37)		
Partially improved	13 (26)	-	7 (20)		
Intact	17 (34)	9 (100)	11 (31)		
Worsened	8 (16)	0 (0)	4 (11)		
Best postoperative visual acuity, median (IQR)	0.7 (0.27–1.0)	1.0 (1.0–1.0)	0.6 (0.16–1.0)		0.77
Time until best visual acuity reached (mo), median (IQR)	4.5 (1.75–12)	-	6 (2.5–19)		0.28
Secondary vision deterioration	10 (20)	2 (22)	12 (34)		0.14
Reoperation due to vision impairment	4 (8)	1 (11)	6 (17)		0.20
	Preoperative impaired visual field	Preoperative intact visual field	Preoperative impaired visual field	Preoperative intact visual field	
	(*n* = 35)	(*n* = 18)	(*n* = 25)	(*n* = 8)	
Postoperative visual field status					< 0.001
Same as preoperative	16 (46)	-	8 (33)	-	
Partially improved	8 (23)	-	8 (33)	-	
Intact	9 (26)	17 (94)	6 (25)	7 (88)	
Worsened	2 (6)	1 (6)	2 (8)	1 (12)	

*IQR*; Interquartile Range, *mo*; Months

The median postoperative visual acuity was 0.5 (IQR 0.09–1.0) in cases treated by skull base trained surgeons and in cases treated by general neurosurgeons also 0.5 (IQR 0.01–0.9). In cases with preoperative visual impairment treated by skull base trained surgeons, 30 (60%) reached an improved to intact vision compared to eighteen (51%) cases in the group treated by general neurosurgeons (*p* = 0.60, Table 3). Best postoperative visual acuity during follow-up was 0.7 (IQR 0.27–1.0) among the cases treated by skull base trained surgeons and 0.6 (IQR 0.16–1.0) among patients treated by general neurosurgeons (*p* = 0.77). The multivariable linear regression analysis showed that cases with higher preoperative visual acuity (β 0.44; 95% CI 0.23–0.66; *p* < 0.001) could achieve a higher maximum postoperative visual acuity (Table 4).

**Table 4:** Multivariable linear regression analysis

	Best postoperative visual acuity			
	β	SE	95% CI	P value
Skull base trained surgeon	0.02	0.09	−0.15–0.19	0.84
Preoperative visual acuity	0.44	0.11	0.23–0.66	< 0.001
Optic canal decompression	−0.098	0.11	−0.33–0.13	0.40
Clinoidectomy	−0.098	0.10	−0.30–0.11	0.35
Simpson Grade—Grade II	0.13	0.09	−0.06–0.32	0.17
Simpson Grade – Grade III	0.06	0.097	−0.14–0.25	0.56
Age	−0.003	0.003	−0.009–0.003	0.26
Lesion location: tuberculum sellae	0.10	0.10	−0.09–0.30	0.30
Lesion location: anterior clinoid process	0.21	0.12	−0.03–0.45	0.08
Lesion location: sphenoid outer-ridge	0.20	0.22	−0.26–0.62	0.42
Hard tumor consistency around the optic nerve	−0.35	0.23	−0.76–0.05	0.09
Soft tumor consistency around the optic nerve	−0.07	0.12	−0.30–0.17	0.58
Low difficulty of tumor resection	0.06	0.09	−0.11–0.24	0.46

*SE*; Standard Error, *CI*; Confidence Interval

Ten (20%) patients from the skull base trained surgeon group with preoperative visual impairment showed secondary vision deterioration, and in four (8%) patients reoperation was needed due to vision impairment (Table 3). In the group treated by general neurosurgeons, twelve (34%) patients experienced secondary vision deterioration, of which six (17%) cases who underwent reoperation due to vision impairment (Table 3).

### Tuberculum sellae and anterior clinoid process meningiomas

Sixty-seven patients harboured tuberculum sellae or ACP meningiomas (Table 1, Supplementary Information Table 1). Forty-two patients were treated by skull base trained surgeons and 25 by general neurosurgeons. Baseline characteristics and surgical outcomes are reported in Supplementary Appendix Table 1 and 2. Of the patients treated by skull base trained surgeons, 36 had preoperative visual impairment and six had intact vision preoperatively (Table 5). The median postoperative visual acuity was 0.5 (IQR 0.1–1.0) in the group treated by skull base trained surgeons and 0.45 (IQR 0.01–1.0) in patients treated by general neurosurgeons (*p* = 0.41). No difference was found in the best postoperative visual acuity between both groups. In cases with preoperative visual impairment treated by skull base trained surgeons, 22 (61%) reached an improved to intact vision compared to fourteen (56%) cases in the group treated by general neurosurgeons (Table 5; *p* = 0.44). Four (11%) patients treated by skull base trained surgeons experienced secondary vision deterioration compared to ten (40%) patients in the group treated by general neurosurgeons (*p* = 0.008).

**Table 5:** Postoperative visual outcomes of patients treated by skull base trained surgeons vs. general neurosurgeons: tuberculum sellae and anterior clinoid process meningiomas

	No. of patients (%)				No. of patients (%)
	Skull base trained surgeon		General neurosurgeon		*P* value
	(*n* = 42)		(*n* = 25)		
	Preoperative impaired vision	Preoperative intact vision	Preoperative impaired vision		
	(*n* = 36)	(*n* = 6)	(*n* = 25)		
Postoperative visual acuity, median (IQR)	0.5 (0.1–1.0)	1.0 (1.0–1.0)	0.45 (0.01–1.0)		0.41
Postoperative visual acuity					0.44
Same as preoperative	9 (25)	-	10 (40)		
Partially improved	9 (25)	-	5 (20)		
Intact	13 (36)	6 (100)	9 (36)		
Worsened	5 (14)	0 (0)	1 (4)		
Best postoperative visual acuity, median (IQR)	0.7 (0.32–1.0)	1.0 (1.0–1.0)	0.67 (0.25–1.0)		0.98
Time until best visual acuity reached (mo), median (IQR)	5 (1–12)	-	6 (1.5–16)		0.74
Secondary vision deterioration	4 (11)	1 (17)	10 (40)		0.008
Reoperation due to vision impairment	1 (3)	1 (17)	4 (16)		0.15
	Preoperative impaired visual field	Preoperative intact visual field	Preoperative impaired visual field	Preoperative intact visual field	
	(*n* = 27)	(*n* = 13)	(*n* = 21)	(*n* = 3)	
Postoperative visual field status					0.01
Same as preoperative	11 (41)	-	6 (30)	-	
Partially improved	7 (26)	-	7 (35)	-	
Intact	7 (26)	13 (100)	6 (30)	3 (100)	
Worsened	2 (7)	0 (0)	1 (5)	0 (0)	

*IQR*; Interquartile Range, *mo*; Months

### Change in postoperative visual acuity for preoperatively severely impaired vision

**Fig. 5:**
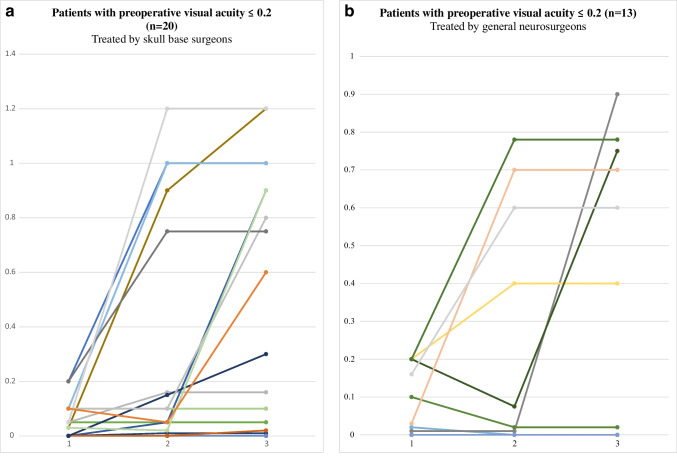
**a** Overview of visual acuity per patients with a preoperative visual acuity of 0.2 or lower – patients treated by skull base surgeons. **b** Overview of visual acuity per patients with a preoperative visual acuity of 0.2 or lower – patients treated by general neurosurgeons

Figures 5a&b display the change in visual acuity of cases with a preoperative visual acuity of 0.2 or lower (35%, *n* = 33) in three different time points: preoperative, postoperative, and the best postoperative visual acuity. Twenty (61%) cases were treated by skull base trained surgeons and thirteen (39%) by general neurosurgeons.

In cases treated by skull base trained surgeons, nine (45%) cases reached a best postoperative visual acuity of 0.5 or higher (Fig. 5a), compared to five (38%) cases treated by general neurosurgeons (Fig. 5b, *p* = 0.41). In five cases (25%) treated by skull base trained surgeons, the postoperative visual acuity initially remained 0.2 or lower, however, the best postoperative visual acuity eventually rose above 0.2, as high as 0.9 (Fig. 5a&b). Two (15%) cases from the group treated by general neurosurgeons showed the same change in visual acuity (*p* = 0.92). In total, 52% of the cases (*n* = 17) experienced significant vision improvement during the follow-up period (Fig. 5a&b).

Nineteen cases (20%) presented with functional blindness preoperatively (visual acuity < 0.1) (Fig. 5a&b). In these cases, 47% (*n* = 9) eventually reached a best postoperative visual acuity of 0.5 or higher (Fig. 5a&b). Seven (78%) of these cases were treated by skull base trained surgeons (Fig. 5a). In cases with postoperative vision improvement, the period of preoperative blindness was five months (IQR 3–7). Cases with no postoperative vision improvement were preoperatively blind for a median of seven months (IQR 3–18; *p* = 0.45).

## Discussion

### Key findings

In a high-volume skull base center, in the past ten years, a total of 709 patients underwent craniotomies for anterior skull base lesions. Of these, 94 patients showed optic nerve compression on MRI with or without preoperative visual impairment. EAC was performed in 53% of the cases. In total, 59 cases were treated by specialised skull base trained surgeons and 35 by general neurosurgeons. There was no significant difference between cases treated by skull base trained surgeons and general neurosurgeons in terms of postoperative permanent complications. In patients with tuberculum sellae or anterior clinoid process meningiomas, postoperative secondary deterioration of visual acuity occurred in 40% (*n* = 10) of the cases treated by general neurosurgeons versus 11% (*n* = 4) in the group treated by skull base trained surgeons (*p* = 0.008). In total, 42% of cases with a preoperative vision of < 0.2 and 47% of those assessed as functionally blind improved to a best postoperative vision of 0.5 or higher. In all meningioma cases in which the optic canal was unroofed, even in cases in which the MRI did not suggest growth into the optic canal, tumor was found encroached around the optic nerve in the canal. Multivariable regression analysis revealed that patients with higher preoperative visual acuity would reach a higher best visual acuity postoperatively.

### Optic canal unroofing as a subspeciality entrusted to skull base trained surgeons

**Fig. 6:**
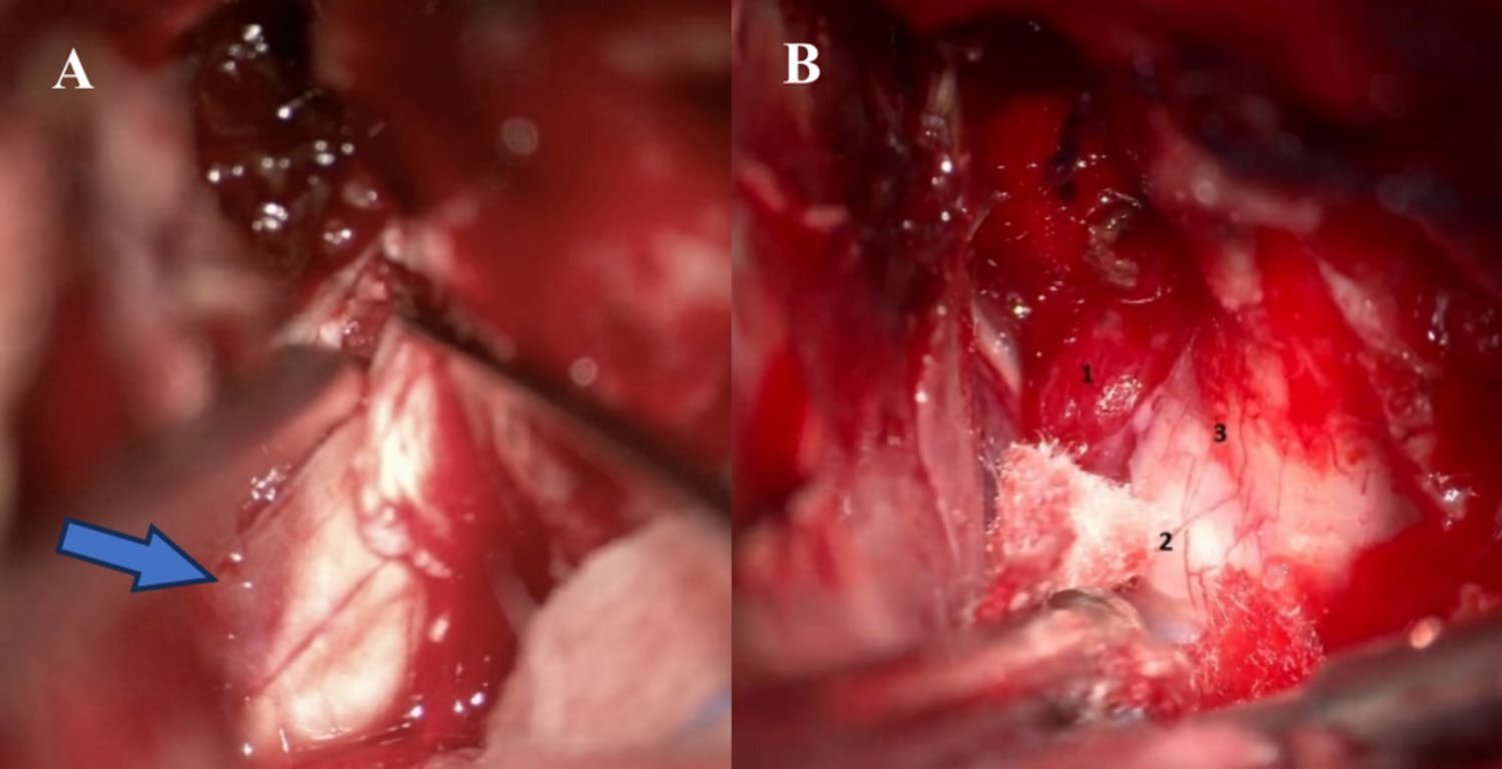
**(A) Perioperative image of optic nerve contusion**. Right-sided optic nerve, note the open canal, opened canal dura overlying the opticus and the tumor medial to the nerve, despite no evidence of canal involvement on MRI. Note the contusion (blue arrow) at the level of the falciform ligament and the meningioma invasion on the medial side of the canal despite the very small size of the meningioma. **(B) **1: Tumor inside the optic canal, 2: optic nerve, 3: contusion optic nerve caused by falciform ligament

According to the training principles in the Netherlands, every neurosurgeon that becomes board certified should possess a broad armamentarium of lesions that he or she can treat. Tuberculum sellae or anterior clinoid meningiomas were traditionally considered lesions not necessarily entrusted to skull base surgeons. The paradigm shift in our institution occurred upon an internal benchmarking assessment in which we noticed that most recurrences occurred in the region or inside the optic canal, even in patients where MRI images did not suggest optic canal involvement. From 2013 onwards, after the first extradural ACP resection was performed in our institution, ACP resection and optic canal unroofing became routine. Completely freeing up the optic nerve allows for more freedom of movement and more potential to displace the nerve, which does not remain tethered to the canal and the falciform ligament. [4] Oftentimes there is a small contusion at the level of the ligament in patients with longstanding compression, which is visible only when the canal is opened (Fig. 6). This, together with the observation that meningiomas with optic nerve involvement virtually always grow inside the canal, has prompted us to centralize these lesions definitively to specialised skull base trained surgeons.

### Visual acuity in patients with severe deterioration preoperatively

Results of patients with severely impaired vision preoperatively (in particular patients who are functionally blind) are scarce in the current scientific literature. Routinely, patients who presented with severely impaired vision, or absent light perception, would sometimes be counselled to receive radiotherapy given that there would be no perceived benefit to be expected postoperatively. Nevertheless, even in patients with absent light perception, at least 47% may recover to a visual acuity higher than 0.5, even as high as 0.9, making any attempt to dissect the optic nerve free and remove tumor highly relevant. Functional blindness, even one of longer duration, does not automatically mean that surgery is not beneficial. Based on our results, future research should focus on evaluating the benefits of surgery for patients with severe impaired preoperative vision, even for patients with long-term preoperative deterioration. Also needed are better imaging markers and prognosticators of potential improvement postoperatively to better identify patients that benefit from this surgery. Last but not least, the finesse of the ultra-microsurgery instruments should be improved to facilitate sharp dissection without vascular injury along the optic nerve.

### The extradural approach to optic nerve unroofing in tuberculum sellae meningiomas

When Dolenc introduced the optic nerve unroofing technique in 1985, this technique was applied for treatment of carotid-ophthalmic aneurysms [8]. In the next decade, this technique was also recognized to be effective and efficient in treatment of supra- and parasellar processes [22]. A systematic review by Lin et al. [11] provides an overview of the current state of effectiveness and safety of optic nerve unroofing for tuberculum sellae meningiomas. Based on the results of fifteen studies, the overall postoperative visual improvement occurred in 79% of the patients (95% Confidence Interval [CI] 70%–87.5%, *p* < 0.01). [11] In our study, in cases with preoperative visual impairment treated by skull base trained surgeons, 22 (61%) reached an improved to intact vision compared to fourteen (56%) cases in the group treated by general neurosurgeons (*p* = 0.44). Postoperative visual deterioration and olfactory nerve damage were the most reported complications. Visual deterioration was reported in nine studies, with a rate of 8% (95% CI 4.1%–11.3%, *p* < 0.01) and olfactory nerve damage was reported in six studies with an overall rate of 5% (95% CI 1.9–9%, *p* < 0.01) [11]. In our subgroup analysis including patients with tuberculum sellae and ACP meningiomas, we found no olfactory nerve damage and a total of six (9%) patients with postoperative visual deterioration compared to preoperative status. The International Tuberculum Sellae Meningioma Study by Magill et al. (2023) included 947 patients harbouring tuberculum sellae meningiomas from 40 institutions, comparing the postoperative visual outcomes between transcranial approach and an expanded endonasal approach [12]. In all patients from both groups, the rate of postoperative visual acuity worsening was 9.8%. [12] Mathiesen et al. reported no patients with postoperative visual deterioration [14]. Chen et al., including one of the largest cohorts in the literature (*n* = 87), reported twelve (14%) cases where visual acuity was worsened postoperatively. [7] However, we found that 40% (*n* = 10) of patients harbouring tuberculum sellae meningiomas treated by general neurosurgeons experienced secondary vision deterioration during postoperative follow-up, whereas in four (16%) a reoperation due to visual impairment was performed. In the skull base trained group, four (11%) patients experienced secondary deterioration, and one patient (3%) was reoperated due to vision impairment (*p* = 0.008). The absolute difference in the incidence of secondary vision deterioration during follow-up is likely due to the skill with which the optic nerve is manipulated. While tumors treated by skull base trained surgeons were more complex, secondary vision deterioration occurred four times less often in this group. To optimize effectiveness and safety of optic nerve unroofing in cases with tuberculum sellae and ACP meningiomas, this technique needs to be performed by expert skull base surgeons in high volume centers, specially trained in performing optic nerve unroofing. One of the reasons for delayed postoperative vision deterioration is vasospasm, which is considered to be a rare finding [2, 6, 18, 21]. In our study, secondary vision deterioration postoperatively occurred during follow-up, which makes the etiology of vasospasm being less likely in these cases. However, in patients with delayed visual acuity worsening in the postoperative course (e.g. two days post-resection), vasospasm should be considered in the differential diagnoses. As the therapeutic window is narrow and optic nerve ischemia could lead to permanent blindness in these cases, postoperative visual acuity should be monitored closely. If necessary, hypertensive therapy and administration of intra-arterial vasoactive agents should be considered in cases with angiography confirmed vasospasm [18].

#### Potential predictors for change in postoperative visual acuity

According to our multivariable linear regression analysis, though underpowered, cases with higher preoperative visual acuity could achieve a higher maximum postoperative visual acuity, which has also been established in previous evidence [12]. However, the vascular supply of the optic nerve is considered to play a significant role in postoperative visual recovery. Cases with postoperative visual decline without evident hematomas suggest pial blood flow deterioration [18]. With use of intraoperative indocyanine green (ICG) angiography before and after resection of tumors with optic apparatus involvement, the optic nerve perfusion can be assessed. [9] Larger multicenter cohorts are necessary to explore the potential of change in intraoperative ICG perfusion as a reliable predictor for postoperative visual acuity improvement. In addition to intraoperative ICG angiography, the Neurological Pupil index (NPi) can be used to evaluate pupil reactivity and findings may correlate closely with visual function, as NPi monitors the proximal portion of the oculomotor reflex [17]. Future studies should focus on investigating the accuracy of NPi as a predictor for change in visual function after resection of tumors compromising the optic apparatus.

### Strengths and limitations

This study is one of the largest cohorts in the literature which investigates the effect of routine optic nerve unroofing in patients with anterior skull base pathologies. As postoperative results of patient with severe (long-term) visual impairment are scarce in the current literature, our study adds a great contribution to the literature for this specific patient population. Upfront radiotherapy in these cases needs to be reconsidered and prognosticators for an improvement in postoperative visual acuity are needed.

However, even with a relatively large cohort compared to the existing literature, the study population of this study remains objectively small. This is mainly due to large number of case exclusions (*n* = 195) due to missing visual acuity measurements (preoperative and/or postoperative), which suggests inconsistency in practice. In our historical cohort, treated by general neurosurgeons, skull base trained surgeons with experience in optic nerve unroofing were also partially involved in the decision-making process, which could explain that we found no difference between the two groups in the multivariable linear regression analysis.

## Conclusion

Patients with tuberculum sellae and anterior clinoid process meningiomas benefit from skull base surgeons trained in extradural optic canal decompression, as reflected by lower postoperative secondary visual acuity deterioration in patients treated by skull base trained surgeons. All cases presenting with tumors with optic apparatus involvement should be managed by skull base trained surgeons to maximize postoperative visual acuity preservation.

## Supplementary information

Below is the link to the electronic supplementary material.

ESM 1 (DOCX 26.9 KB)

## Data Availability

Anonymized data is available upon reasonable written request to the corresponding author.

## References

[1] Al-Mefty O (1990) Clinoidal meningiomas. J Neurosurg 73(6):840–849 2230967 10.3171/jns.1990.73.6.0840

[2] Aoki N, Origitano TC, al-Mefty O (1995) Vasospasm after resection of skull base tumors. Acta Neurochir (Wien) 132(1–3):53–58. 10.1007/BF01404848 10.1007/BF01404848 7754859

[3] Basma J, Moore KA, Krisht K, Abuelem T, Arnautovic K, Michael LM, Aboud E, Krisht AF (2020) Morphometric comparison of the pterional trans-sylvian and the pretemporal trans-clinoidal approaches to the posterior communicating artery. Oper Neurosurg (Hagerstown) 20(1):E22–E30 32860710 10.1093/ons/opaa261

[4] Basma J, Dacus MR, Kumar R, Spencer D, Arnautović KI (2023) Cisternal, falciform, and optic canal decompression influencing optic nerve biomechanics: a microsurgical anatomic study. Oper Neurosurg (Hagerstown) 24(2):e75–e84 36637310 10.1227/ons.0000000000000472

[5] Baucher G, Troude L, Roux A et al (2022) Predictors of visual function after resection of skull base meningiomas with extradural anterior clinoidectomy. Neurosurg Rev 45(3):2133–2149 35006456 10.1007/s10143-021-01716-w

[6] Bejjani GK, Sekhar LN, Yost AM, Bank WO, Wright DC (1999) Vasospasm after cranial base tumor resection: pathogenesis, diagnosis, and therapy. Surg Neurol 52(6):577–584. 10.1016/s0090-3019(99)00108-1 10660023 10.1016/s0090-3019(99)00108-1

[7] Chen L, Gao M, Zhang H, Chen W, Sun K, Xu R (2024) Effect of optic canal opening on postoperative visual acuity in patients with tuberculum sellae meningiomas. J Neurol Surg A Cent Eur Neurosurg 85(1):1–6. 10.1055/a-1768-3553 35144298 10.1055/a-1768-3553

[8] Dolenc VV (1985) A combined epi- and subdural direct approach to carotid-ophthalmic artery aneurysms. J Neurosurg 62(5):667–672 3989589 10.3171/jns.1985.62.5.0667

[9] Han SJ, Magill ST, Tarapore PE, Horton JC, McDermott MW (2016) Direct visualization of improved optic nerve pial vascular supply following tuberculum meningioma resection: case report. J Neurosurg 125(3):565–569. 10.3171/2015.6.JNS15765 26684783 10.3171/2015.6.JNS15765 PMC5125447

[10] Lehmberg J, Krieg SM, Mueller B, Meyer B (2013) Impact of anterior clinoidectomy on visual function after resection of meningiomas in and around the optic canal. Acta Neurochir (Wien) 155(7):1293–1299 23665725 10.1007/s00701-013-1741-x PMC3683144

[11] Lin PW, You W, Guo AS, Lin ZR, Wang YZ (2023) Efficiency and safety of optic canal unroofing in tuberculum sellae meningiomas: a meta-analysis and systematic review. Neurosurg Rev. 46(1):240 37698750 10.1007/s10143-023-02151-9 PMC10497650

[12] Magill ST, Schwartz TH, Couldwell WT et al (2023) International tuberculum sellae meningioma study: surgical outcomes and management trends. Neurosurgery 93(6):1259–1270. 10.1227/neu.0000000000002569 37389475 10.1227/neu.0000000000002569 PMC12245236

[13] Mariniello G, de Divitiis O, Bonavolontà G, Maiuri F (2013) Surgical unroofing of the optic canal and visual outcome in basal meningiomas. Acta Neurochir (Wien) 155(1):77–84 (**Erratum in: Acta Neurochir (Wien). 2013;155(1):85–6**) 22945895 10.1007/s00701-012-1485-z

[14] Mathiesen T, Kihlström L (2006) Visual outcome of tuberculum sellae meningiomas after extradural optic nerve decompression. Neurosurgery 59(3):570–576. 10.1227/01.NEU.0000228683.79123.F9. (**discussion 570–6**) 16955039 10.1227/01.NEU.0000228683.79123.F9

[15] Noguchi A, Balasingam V, Shiokawa Y, McMenomey SO, Delashaw JB Jr (2005) Extradural anterior clinoidectomy. Technical note. J Neurosurg 102(5):945–950 15926728 10.3171/jns.2005.102.5.0945

[16] Otani N, Muroi C, Yano H, Khan N, Pangalu A, Yonekawa Y (2006) Surgical management of tuberculum sellae meningioma: role of selective extradural anterior clinoidectomy. Br J Neurosurg 20(3):129–138 16801044 10.1080/02688690600776747

[17] Raygor KP, Theodosopoulos PV (2019) Use of the neurological pupil index to predict postoperative visual function after resection of a tuberculum sellae meningioma: a case report. Cureus 11(10):e5998. 10.7759/cureus.5998 31807386 10.7759/cureus.5998 PMC6876898

[18] Santarius T, Jian BJ, Englot D, McDermott MW (2014) Delayed neurological deficit following resection of tuberculum sellae meningioma: report of two cases, one with permanent and one with reversible visual impairment. Acta Neurochir (Wien) 156(6):1099–1102. 10.1007/s00701-014-2046-4 24639145 10.1007/s00701-014-2046-4

[19] Sharma A, Rieth GE, Tanenbaum JE, Williams JS, Ota N, Chakravarthi S, Manjila S, Kassam A, Yapicilar B (2018) A morphometric survey of the parasellar region in more than 2700 skulls: emphasis on the middle clinoid process variants and implications in endoscopic and microsurgical approaches. J Neurosurg 129(1):60–70 28799880 10.3171/2017.2.JNS162114

[20] Suprasanna K, Ravikiran SR, Kumar A, Chavadi C, Pulastya S (2015) Optic strut and para-clinoid region - assessment by multi-detector computed tomography with multiplanar and 3 dimensional reconstructions. J Clin Diagn Res 9(10):TC06-9 26557589 10.7860/JCDR/2015/15698.6615 PMC4625308

[21] Taussky P, Kalra R, Couldwell WT (2012) Delayed vasospasm after removal of a skull base meningioma. J Neurol Surg A Cent Eur Neurosurg 73(4):249–252. 10.1055/s-0032-1313589 21104584 10.1055/s-0032-1313589

[22] Yonekawa Y, Ogata N, Imhof HG et al (1997) Selective extradural anterior clinoidectomy for supra- and parasellar processes. Technical note. J Neurosurg 87(4):636–642 9322855 10.3171/jns.1997.87.4.0636

