# Supplementary material for: Effect of routine extradural optic canal decompression performed by skull base trained surgeons on visual outcomes in patients with anterior skull base meningiomas
Source: Acta Neurochir (Wien). 2025 Jun 16;167(1):170. doi: 10.1007/s00701-025-06584-7 (PMC12170688; doi:10.1007/s00701-025-06584-7)
Supplement: Supplementary file 1 — (DOCX 26.9 KB) [file 701_2025_6584_MOESM1_ESM.docx]

**Supplementary Information**

**Effect of Routine Extradural Optic Canal Decompression on Visual Outcomes in Patients with Anterior Skull Base Meningiomas**

**Acta Neurochirurgica**

Yasmin Sadigh, MSc^1,2^, Lailla Talbi, MSc^1^*, Juliette Monchen, MSc^1^*, Ayca Cozar, MSc^1^**, Kelsey Gori, MSc^1^**, Ruben Dammers, MD, PhD^1,2#^, Victor Volovici, MD, PhD^1,2#^

*^1^Department of Neurosurgery, Erasmus MC Stroke Center, Erasmus MC University Medical Centre, Rotterdam, The Netherlands**.*

*^2^Centre* *for Complex Microvascular Surgery, Erasmus MC University Medical Centre, Rotterdam, The Netherlands.*

***** = these authors contributed equally.

** = these authors contributed equally.

# = These authors contributed equally.

Corresponding author:

Victor Volovici, MD, PhD

Departments of Neurosurgery, Erasmus MC Stroke Center

Center for Complex Microvascular Surgery

Erasmus MC University Medical Center

Dr Molewaterplein 40

3015 GD

Rotterdam, The Netherlands

Phone: 0031107040704

E-mail: v.volovici@erasmusmc.nl

Supplementary Appendix Table 1. Baseline characteristics of patients treated by skull base trained surgeons vs. general neurosurgeons: tuberculum sellae and anterior clinoid process meningiomas

Supplementary Appendix Table 2. Surgical outcomes of cases treated by skull base trained surgeons vs. general neurosurgeons: tuberculum sellae and anterior clinoid process meningiomas

| **Supplementary Appendix Table 1. Baseline characteristics of patients treated by skull base trained surgeons vs. general neurosurgeons: tuberculum sellae and anterior clinoid process meningiomas** | | | | | |
| --- | --- | --- | --- | --- | --- |
|  | | **No. of patients (%)** | | | |
|  |  | **All patients** | **Skull base trained surgeon** | **General neurosurgeon** | **P value** |
|  |  | **(n=67)** | **(n=42)** | **(n=25)** |  |
| Sex, female | | 52 (78) | 33 (79) | 19 (76) | 0.81 |
| Age (year), median (IQR) | | 52 (42-63) | 51 (38.7-63) | 52 (46-65) | 0.54 |
| Duration vision impairment (mo), median (IQR) | | 7 (3-12) | 6 (2-12) | 7 (3.7-19.5) | 0.38 |
| Preoperative visual field | | | | | 0.17 |
|  | Intact | 16 (25) | 13 (32) | 3 (12) |  |
|  | Homonymous hemianopia | 6 (9) | 5 (12) | 1 (4) |  |
|  | Bitemporal hemianopia | 9 (14) | 5 (12) | 4 (17) |  |
|  | Quadrantanopia superior | 2 (3) | 1 (2) | 1 (4) |  |
|  | Quadrantanopia inferior | 7 (11) | 3 (7) | 4 (17) |  |
|  | Nasal hemianopsia | 3 (5) | 3 (7) | 0 (0) |  |
|  | Non-intact (not specified) | 21 (33) | 10 (25) | 11 (46) |  |
| Preoperative visual acuity, median (IQR) | | 0.4 (0.1-0.8) | 0.4 (0.1-0.9) | 0.25 (0.06-0.55) | 0.16 |
| Lesion location | | | | | 0.17 |
|  | Tuberculum sellae | 44 (66) | 25 (59) | 19 (76) |  |
|  | Anterior clinoid process | 23 (34) | 17 (40) | 6 (24) |  |
| Lesion size (mm), median (IQR) | | 27 (20.5-37.5) | 29 (19.7-38) | 26 (21.7-33) | 0.90 |

**IQR: Interquartile Range, mo: Months.**

| **Supplementary Appendix Table 2. Surgical outcomes of patients treated by skull base trained surgeons vs. general neurosurgeons: tuberculum sellae and anterior clinoid process meningiomas** | | | | | |
| --- | --- | --- | --- | --- | --- |
|  | | **No. of patients (%)** | | | |
|  |  | **All patients** | **Skull base trained surgeon** | **General neurosurgeon** | **P value** |
|  |  | **(n=67)** | **(n=42)** | **(n=25)** |  |
| Surgical approach | | | | | 0.87 |
|  | Pterional | 50 (78) | 32 (76) | 18 (82) |  |
|  | Pretemporal | 8 (12) | 6 (14) | 2 (9) |  |
|  | Fronto-orbital | 4 (6) | 2 (5) | 2 (9) |  |
|  | Anterior petrosectomy | 1 (2) | 1 (2) | 0 (0) |  |
|  | Trans-sphenoidal | 1 (1) | 1 (2) | 0 (0) |  |
| Optic canal decompression | | 49 (75) | 30 (73) | 19 (79) | 0.59 |
| Clinoidectomy | | 38 (59) | 25 (61) | 13 (56) | 0.73 |
| Simpson Grade Meningioma | | | | | 0.73 |
|  | Grade 0 | 2 (3) | 2 (5) | 0 (0) |  |
|  | Grade I | 2 (3) | 2 (5) | 0 (0) |  |
|  | Grade II | 27 (42) | 18 (44) | 9 (39) |  |
|  | Grade III | 17 (27) | 10 (24) | 7 (30) |  |
|  | Grade IV | 16 (25) | 9 (22) | 7 (30) |  |
|  | Missing Simpson Grade | 3 | 1 | 2 |  |
| Postoperative transient complications | | 12 (18) | 4 (9) | 8 (32) | 0.04 |
| Postoperative permanent complications | | 5 (8) | 4 (10) | 1 (4) | 0.64 |
| Meningioma WHO Tumor Grade | | | | | 0.63 |
|  | Grade I | 63 (94) | 40 (95) | 23 (92) |  |
|  | Grade II | 4 (6) | 2 (5) | 2 (8) |  |
| Postoperative radiotherapy | | 1 (1) | 0 (0) | 1 (4) | 0.37 |
| No. of recurrences | | 5 (7) | 1 (2) | 4 (16) | 0.06 |
| Time until recurrence (mo), median (IQR) | | 36 (25-70) | 36 (36-36) | 45 (19.5-77.5) | 0.72 |

**IQR: Interquartile Range, mo: Months, WHO: World Health Organization.**
